# Correlations between physical activity and neurocognitive domain functions in patients with schizophrenia: a cross-sectional study

**DOI:** 10.1186/s12888-016-1176-z

**Published:** 2017-01-05

**Authors:** Yusuke Kurebayashi, Junichi Otaki

**Affiliations:** 1 Faculty of Nursing, Kansai University of Health Science, 2-11-1 Wakaba, Kumatori, Sennan, Osaka Japan; 2 Graduate School of Health Science, Kyorin University, 476 Miyashia cho, Hachioji City, Tokyo Japan

**Keywords:** Schizophrenia, Physical activity, Neurocognitive function, Inpatients, Non-remission, Treatment-resistance

## Abstract

**Background:**

Neurocognitive dysfunction is a critical target symptom of schizophrenia treatment. A positive correlation between physical activity level and neurocognitive function has been reported in healthy individuals, but it is unclear whether such a correlation exists in patients with schizophrenia and whether the relationship is different according to inpatients or outpatients. This study aimed to examine the differences in the correlations between physical activity and multiple neurocognitive domains in inpatients and outpatients with schizophrenia and obtain suggestions for further study to facilitate this field.

**Methods:**

Twenty-nine patients with schizophrenia were examined (16 inpatients and 13 outpatients, 56.0 ± 11.4 years of age). Current symptoms were assessed using the Positive and Negative Symptom Scale and neurocognitive functions using Cognitrax, which yields a composite neurocognitive index (NCI) and 11 domain scores. After testing, participants wore an HJA-750C accelerometer for one week to measure physical activity levels and durations. Partial correlation analyses were performed between exercise and cognitive parameters.

**Results:**

In the outpatient group, higher physical activity was associated with faster Motor and Psychomotor Speeds in outpatients. However, higher physical activity was associated with lower overall NCI, Attention score, and Memory scores in inpatients.

**Conclusion:**

Although higher physical activity was associated with better neurocognitive functions of outpatients, in inpatients with non-remitted schizophrenia, higher physical activity was associated with worsening of several cognitive domains. In a future study examining the relationship between physical activity and neurocognitive function for facilitating this research field, separation between inpatients and outpatients are needed because the relationship is different between inpatients and outpatients.

**Electronic supplementary material:**

The online version of this article (doi:10.1186/s12888-016-1176-z) contains supplementary material, which is available to authorized users.

## Background

In addition to prominent positive and negative symptoms, several patients with schizophrenia have neurocognitive deficits. The severity of these deficits is a strong predictor of poor social functioning [1] and issues in daily life [2]; therefore, cognitive dysfunction is recognised as a core feature of schizophrenia, and comprehensive treatment must include improvement or maintenance of cognitive functioning and mitigation of positive and negative symptoms.

In healthy individuals, physical activity can improve cognitive function. A large 26-year cohort study [3] reported that healthy people who routinely exercised showed better cognitive performance than sedentary people. A meta-analysis by Hidin et al. [4] of studies involving healthy adults ≥55 years concluded that physical activity improves cognitive function. In addition, Smith et al. [5] conducted a systematic review of 29 randomised controlled trails (RCTs) and concluded that aerobic activity enhances cognitive function in healthy adults.

On the other hand, the effects of physical activity on neurocognitive functions in clinical populations are yet unclear. A recent review [6, 7] stated that such studies have neglected the effects of physical exercise on cognitive performance in patients with schizophrenia despite the strong association between cognitive function and clinical outcome. Pajonk et al. [8] conducted an RCT including 24 schizophrenia outpatients and reported improved memory and large hippocampal volume in active patients compared with the non-exercising patient group. Kimhy et al. [9] reported that maximum oxygen consumption, one indicator of physical activity level, was positively correlated with cognitive function in 32 schizophrenia outpatients. Similarly, Leutwyler et al. [10] reported a positive correlation between physical activity level and Processing Speed in schizophrenia outpatients.

However, several issues are yet unclear. First, the correlation between cognitive function and physical activity has not been investigated in inpatients with schizophrenia. If inpatients were included in a study, only the combing score of inpatients and outpatients was analysed. No subgroup analysis of inpatients and outpatients was performed. Because several factors influence cognitive function differently based on whether the disease is well controlled or active (in outpatients and inpatients, respectively) [11], the effects of physical activity may differ. To facilitate the study field, subgroup analysis of inpatients and outpatients is needed. Second, most studies have reported only one or a few measures; however, cognitive function consists of multiple elements or neurocognitive domains. The MATRICS Consensus Cognitive Battery includes 7 domains that are considered necessary for a complete evaluation of cognitive functioning in schizophrenia [12]. However, most previous studies have only investigated the correlation between physical activity and memory or general cognitive function [13].

Clarifying the correlations between physical activity and the neurocognitive domains affected in schizophrenia could facilitate decisions regarding treatment and hospital discharge, which would relieve the burden on institutions with limited capacity. This preliminary study aimed to examine the correlations between physical activity levels and multiple neurocognitive domains as measured using the Cognitrax testing system in patients with schizophrenia. It also aimed to examine differences in these correlations according to the treatment site (inpatient vs. outpatient) and to obtain suggestions for further study to facilitate this field.

## Methods

### Study design

This was a cross-sectional study including 29 patients diagnosed with schizophrenia (enrolled from two centres in Osaka, Japan).

### Participants

All participants were recruited from closed psychiatric hospital wards or day care centres. Inclusion criteria were as follows: (1) diagnosis of schizophrenia by their doctors, (2) consent from both the patient and treating physician, (3) voluntary treatment at a day care centre for outpatients and (4) voluntary hospitalization according to Japanese law for inpatients. To enhance the comparability between inpatients and outpatients, we included inpatients under voluntary hospitalization. For example, there may be the bias that the data of inpatients under non-voluntary hospitalization tended to have more differences with the data of outpatients compared with the data of inpatients under voluntary hospitalization. Exclusion criteria were as follows: (1) severe psychiatric symptoms such as those requiring seclusion or body restraint, (2) no dependent gait, (3) <20 years old, (4) treated using lobotomy.

### Measurements

#### Demographics

Demographic and baseline clinical information (age, sex, age of onset, duration of illness, length of stay, body mass index (BMI), blood pressure, education, and the category and dose of medications) was collected from medical records. The dose of antipsychotics was calculated by chlorpromazine (CP) equivalents, antianxiety medication by diazepam equivalents, and anti-Parkinsonian medication by biperiden equivalents.

#### Psychiatric symptoms

The Positive and Negative Syndrome Scale (PANSS), consisting of positive, negative, and general psychopathology scales, was used to assess current psychiatric symptoms. The validity and reliability of PANSS has been demonstrated [14]. All 30 items were rated on a scale from 1 (absent) to 7 (severe) based on symptom severity over a week. The 3 subscale scores and total score on PANSS were used in analysis.

Remission was defined according to Andreasen et al. [15] as scores of ≤3 for delusions (P1), conceptual disorganization (P2) hallucinatory behaviour (P3), blunted affect (N1), passive apathetic social withdrawal (N4), lack of spontaneity and flow of conversation (N6), mannerisms and posturing (G5) and unusual thought content (G9).

We evaluated all psychiatric symptom scores on PANSS according to instructions from trained psychiatrists.

#### Neurocognitive functions

The Cognitrax basic package has been extensively evaluated for reliability and validity [16] and was used to examine neurocognitive functions. The participants performed seven tasks displayed on a personal computer: Verbal Memory, Visual Memory, Finger Tapping, Symbol Digit Cording, Stroop Test, Shifting Attention and Continuous Performance Test. Based on the scores on the 7 tasks, a composite Neurocognition Index (NCI) and 11 Domain Scores were calculated (Composite Memory, Verbal Memory, Visual Memory, Psychomotor Speed, Reaction Time, Complex Attention, Cognitive Flexibility, Processing Speed, Executive Function, Simple Attention and Motor Speed). Individual scores were standardised by setting the mean score to 100 and the standard deviation (SD) to 15; therefore, the scores in participants can be compared those in healthy people without investigating healthy people [16]. Higher score indicates better neurocognitive function.

#### Physical activity

The participants wore a triaxial accelerometer (Active style pro HJA-750C; Omron Colin Co., Ltd., Kyoto, Japan) at the waist for 1 week to measure physical activity. HJA-750C can record METs (metabolic equivalents), EX (Exercise: >3 MET × minutes), total steps per day, total time for step, total calorie consumption, and the daily minutes of physical activity categorized as sedentary (<3.0 METs), moderate (3–5.9METs), and vigorous (≥6.0 METs). All activity indicators used for analysis were calculated as average per day for more than week. These indicators were previously validated [17].

We instructed participants to wear HJA-750C from waking until they slept, for more than 6 h per day, except during showers and baths. To do this, a leaflet which demonstrated adequate wearing was used. To ensure adequate measurement of physical activity, ward nurses instructed the participants on proper wearing of the HJA-750C.

### Procedures

All data were collected from Sep 2015 to Mar 2016. Interviews for measuring psychiatric symptoms and neurocognitive functions were conducted in a private meeting room on the ward. First, we conducted the interview to measure psychiatric symptoms. Next, neurocognitive functions were examined via PC. Finally, immediately after measuring neurocognitive functions, we requested the participants to wear the HJA-750C for 1 week. All psychometric tests were conducted on one day per participant.

### Statistical analysis

The participants were divided into 2 groups: the inpatients group and the outpatients group. The following statistical analyses were performed separately with the inpatients and outpatients groups. First, in all statistical analysis, parametric tests were chosen because nonparametric analysis induces type II errors. The parameters measured in this study were considered to have normal distributions in other studies [16, 18], and recently, parametric analyses have been used except for data with extreme deviations from normal distributions [19]. Furthermore, the correlation of the absolute value was important to the aim of this study, not of rank order in these participants. Therefore, parametric analyses were introduced in this study.

Pearson coefficients were calculated to determine the correlations of demographic variables and psychiatric symptoms with neurocognitive functions (NCI and the 11 domain scores). Second, partial correlation analyses were performed to determine the statistical associations between physical activity and neurocognitive functions (NCI and the 11 domain scores), with controlling for confounder variables that correlated with neurocognitive functions (e.g., age). The partial correlation in the inpatient group was compared with that in the outpatient group.

Using IBM SPSS Sample Power version 3, we estimated that 26 participants were required for a power of 0.8 at alpha = 0.05 assuming a correlation coefficient of 0.5 [10]. Values of *p* < 0.05 were considered significant. All statistical analyses were performed using SPSS for Windows version 23.0.

### Ethical consideration

Participants and their doctors were informed of the study aims and methods using a leaflet. Patients were free to participate or refuse without consequences. All participants and their doctors provided written consent to the study. We asked their doctors to estimate whether participation of patients might affect the state of the patients or not. Then, if the doctor thought that there would be no effect, they gave written consent for the patient in the study. Because individuals who were younger than 20 years old are considered to be minors according to the Japanese law, for grantee voluntary participate, they were exclusive. The research protocol was approved by the ethics committee of Kansai University of Health Science before beginning the investigation.

## Results

All measurements of all participants (16 inpatients and 13 outpatients) were investigated in this study. Demographic features, mean psychometric scores and accelerometry values are shown in Table 1. None of the inpatients were in remission according to the criteria of Andreasen et al. [15], whereas 3 outpatients were in remission.

**Table 1:** Mean scores of participants (*n* = 29)

	Inpatients (*n* = 16)	Outpatients (*n* = 13)
	mean	mean
Demographics		
Age	59.4 ± 12.1	51.8 ± 9.3
Age of onset	27.9 ± 13.5	29.2 ± 9.2
Duration of illness (year)	31.6 ± 14.2	22.6 ± 9.8
Length of stay (month)	207.0 ± 175.9	22.4 ± 46.3
BMI	23.2 ± 2.6	25.1 ± 5.6
Dosage of FGA ( mg )	240.8 ± 334.4	49.0 ± 110.2
Dosage of SGA ( mg )	944.2 ± 700.0	851.6 ± 504.9
Sum dosage of antipsychotics ( mg )	1185.0 ± 890.4	900.6 ± 585.5
Biperiden ( mg )	3.1 ± 3.9	1.3 ± 1.5
Diazepam ( mg )	15.5 ± 14.0	14.0 ± 11.9
Psychiatric symptoms		
Positive scale	14.1 ± 4.9	11.5 ± 3.7
Negative scale	25.7 ± 5.8	16.2 ± 5.6
General psychopathology scale	33.9 ± 6.5	24.2 ± 4.0
PANSS	73.6 ± 13.3	51.9 ± 8.5
Neurocognitive function		
Neurocognitive Index	56.1 ± 21.6	71.0 ± 17.3
Composite Memory	58.7 ± 26.7	67.8 ± 29.1
Verbal Memory	64.6 ± 26.7	74.4 ± 23.4
Visual Memory	68.4 ± 20.2	85.6 ± 20.4
Psychomotor Speed	65.8 ± 17.2	80.8 ± 18.6
Reaction Time	69.8 ± 36.1	59.4 ± 17.0
Complex Attention	34.0 ± 56.6	75.2 ± 25.6
Cognitivie Flexibility	49.8 ± 21.5	64.6 ± 21.6
Processing Speed	71.4 ± 19.2	84.8 ± 15.3
Executive Function	51.4 ± 18.2	65.1 ± 20.8
Simple Attention	−6.9 ± 167.2	93.7 ± 26.0
Motor Speed	72.7 ± 18.3	84.8 ± 18.7
Physical activity		
Calory consumption of walking (kcal)	157.3 ± 181.2	218.4 ± 239.3
Calory consumption of daily activity (kcal)	288.5 ± 129.0	314.5 ± 161.5
Calory consumption of activity (kcal)	445.8 ± 288.1	533.0 ± 314.9
Total calory consumption (kcal)	2013.3 ± 417.8	2064.1 ± 527.3
Ex of walking	2.0 ± 3.0	3.2 ± 3.8
Ex of daily activity	1.2 ± 0.8	1.9 ± 1.3
Total Ex	3.1 ± 3.6	5.1 ± 4.1
Steps	5398.1 ± 6011.7	6821.6 ± 6667.9
Times of walking (min / a day)	69.3 ± 62.7	87.8 ± 71.4
Spending time of sedentary (1 ~ 2.9METS)	662.7 ± 248.4	499.3 ± 189.7
Spending time of moderate (3 ~ 5.9METS)	47.0 ± 59.6	76.4 ± 56.2
Spending time of vigorous (6 ~ 8.9METS)	2.8 ± 8.3	1.6 ± 2.7

### Correlational analyses in inpatients

In inpatients, the raw correlational analyses among demographics, psychiatric symptoms, and neurocognitive functions are shown in the Additional file 1: Table S1. The partial correlation analyses revealed that the total NCI, Complex Attention domain score, and Simple Attention domain score were negatively correlated with physical activity (Table 2). Furthermore, Composite Memory, Verbal Memory, and Visual Memory domain scores were negatively correlated with the time spent in vigorous exercise (>6 METs).

**Table 2:** Parcial Correlation Between Neurocognitive Function and Physical Activity in inpatients (*n* = 16)

	Neurocognitive function																							
Physical activity	Neurocogntiive Index		Composite Memory		Verbal Memory		Visual Memory		Psychomotor Speed		Reaction Time		Complex Attention		Cognitive Flexibility		Processing Speed		Executive Function		Simple Attention		Motor Speed	
	r	p	r	p	r	p	r	p	r	p	r	p	r	p	r	p	r	p	r	p	r	p	r	p
Calory consumption of walking (kcal)	-.507	.064	-.083	.761	.005	.985	-.135	.617	.309	.262	-.327	.234	-.674	.006**	-.330	.213	-.341	.233	-.364	.166	-.630	.021*	.300	.259
Calory consumption of daily activity (kcal)	-.620	.018*	-.316	.233	-.202	.454	-.380	.146	-.083	.768	-.330	.229	-.704	.003**	-.224	.404	-.477	.084	-.227	.398	-.787	.001**	.128	.637
Calory consumption of activity (kcal)	-.593	.025*	-.194	.472	-.087	.749	-.256	.340	.151	.591	-.351	.199	-.730	.002**	-.308	.246	-.424	.131	-.331	.211	-.757	.003**	.246	.359
Total calory consumption (kcal)	-.281	.331	-.110	.686	.085	.753	-.307	.247	.328	.233	-.162	.565	-.474	.074	-.144	.594	-.252	.384	-.176	.515	-.434	.139	.372	.156
Ex of walking	-.568	.034*	-.174	.519	-.070	.796	-.232	.388	.280	.312	-.314	.254	-.714	.003**	-.383	.143	-.332	.246	-.426	.100	-.654	.015*	.244	.362
Ex of daily activity	-.723	.003**	-.117	.665	-.100	.713	-.093	.732	.008	.978	-.284	.304	-.868	.000***	-.381	.145	-.551	.041*	-.328	.215	-.804	.001**	.133	.623
Total Ex	-.629	.016*	-.169	.531	-.080	.767	-.210	.434	.227	.415	-.323	.240	-.784	.001**	-.401	.124	-.401	.156	-.423	.102	-.724	.005**	.230	.392
Steps	-.546	.044*	.011	.968	.081	.765	-.051	.851	.233	.404	-.371	.174	-.723	.002**	-.320	.227	-.406	.150	-.327	.216	-.678	.011*	.252	.347
Times of walking (min)	-.477	.084	.103	.704	.141	.603	.068	.801	.230	.409	-.384	.157	-.644	.010*	-.272	.308	-.395	.162	-.280	.294	-.604	.029*	.269	.313
Spending time of sedentary(1 ~ 2.9METs)	-.406	.150	-.304	.253	-.241	.369	-.340	.198	-.425	.115	-.415	.124	-.339	.217	.020	.942	-.481	.082	.039	.885	-.465	.109	-.063	.818
Spending time of moderate(3 ~ 5.9METs)	-.609	.021*	-.006	.983	.065	.810	-.083	.759	.128	.648	-.365	.181	-.782	.001**	-.322	.224	-.472	.089	-.336	.204	-.771	.002**	.141	.602
Spending time of vigorous(6 ~ 8.9METs)	-.459	.099	-.655	.006**	-.589	.016*	-.503	.047*	.428	.112	.143	.611	-.115	.683	-.287	.282	.197	.500	-.324	.221	-.017	.955	.389	.137

* *p* < 0.05

** *p* < 0.01

*** *p* < 0.001

### Correlational analysis in outpatients

In outpatients, the raw correlational analyses among demographics, psychiatric symptoms, and neurocognitive functions are shown in the Additional file 1: Table S2. The partial correlation analyses revealed that the Psychomotor Speed and Motor Speed domains were positively correlated with physical activity (Table 3).

**Table 3:** Parcial correlation between nuerocognitive function and physical activity in outpatients (*n* = 13)

	Neurocognitive function																							
Physical activity	Neurocogntiive Index		Composite Memory		Verbal Memory		Visual Memory		Psychomotor Speed		Reaction Time		Complex Attention		Cognitive Flexibility		Processing Speed		Executive Function		Simple Attention		Motor Speed	
	r	p	r	p	r	p	r	p	r	p	r	p	r	p	r	p	r	p	r	p	r	p	r	p
Calory consumption of walking (kcal)	.164	.610	.002	.995	-.473	.120	-.216	.478	.566	.044*	.445	.147	.006	.986	.353	.237	.095	.758	.415	.158	-.135	.675	.644	.018*
Calory consumption of daily activity (kcal)	.259	.417	.346	.246	.368	.239	.296	.326	.392	.185	.261	.413	.096	.768	.191	.532	.259	.393	.230	.449	-.354	.260	.400	.176
Calory consumption of activity (kcal)	.262	.411	.179	.558	-.170	.596	-.013	.967	.631	.021*	.473	.121	.054	.868	.366	.219	.205	.501	.434	.139	-.288	.364	.694	.008**
Total calory consumption (kcal)	.293	.355	.123	.690	-.193	.547	-.188	.539	.548	.052	.537	.072	.097	.764	.395	.182	.231	.448	.471	.104	-.180	.576	.576	.040*
Ex of walking	.100	.756	-.043	.890	-.558	.059	-.270	.372	.490	.089	.391	.209	.001	.998	.332	.267	.052	.866	.383	.196	-.046	.887	.562	.046*
Ex of daily activity	.250	.433	.262	.387	.303	.338	.227	.456	.463	.111	.237	.458	.013	.968	.165	.591	.316	.292	.215	.481	-.271	.395	.468	.107
Total Ex	.175	.587	.042	.892	-.428	.166	-.181	.554	.600	.030*	.439	.153	.005	.988	.361	.226	.147	.632	.424	.149	-.129	.689	.669	.012*
Steps	.168	.602	.063	.838	-.341	.278	-.049	.873	.486	.092	.249	.436	-.058	.858	.264	.384	.058	.850	.316	.293	-.199	.534	.569	.043*
Times of walking (min)	.130	.687	.070	.821	-.355	.257	-.089	.774	.566	.044*	.307	.332	-.089	.783	.259	.392	.069	.824	.322	.283	-.242	.449	.668	.013*
Spending time of sedentary(1 ~ 2.9METs)	.152	.638	.409	.166	.443	.149	.442	.131	-.071	.818	.042	.897	.329	.297	.079	.798	-.053	.863	.056	.856	-.434	.159	-.065	.832
Spending time of moderate(3 ~ 5.9METs)	.192	.549	.075	.808	-.367	.240	-.142	.644	.626	.022*	.408	.188	-.012	.970	.352	.238	.175	.568	.416	.158	-.156	.628	.692	.009**
Spending time of vigorous(6 ~ 8.9METs)	.236	.460	.023	.940	-.473	.120	-.142	.643	.602	.029*	.551	.064	.096	.766	.491	.088	.194	.526	.530	.062	.008	.981	.648	.017*

* *p* < 0.05

** *p* < 0.01

*** *p* < 0.001

## Discussion

This pilot study examining the correlations between physical activity levels and multiple neurocognitive domains revealed that the pattern of correlation differed according to neurocognitive function and treatment site. In outpatients, higher physical activity was associated with faster Psychomotor and Motor Speeds but not with Attentional and Memory domain scores. In inpatients, higher physical activity was associated with lower Neurocognitive Index, Complex Attention domain score, Simple Attention domain score, and Memory domain scores.

Leutwyler et al. [10] reported a positive correlation between motor speed and total calorie consumption but no correlations with other neurocognitive domains in 30 patients with schizophrenia. Although a positive relationship between physical activity and motor speed is consistent with our results in outpatients (Table 3), we found a strong negative correlation between physical activity and attentional function in inpatients, but not in outpatients (Tables 2 and 3). Thus, physical activity appears to have distinct relationships with different neurocognitive domains. Furthermore, the correlation pattern between physical activity and neurocognitive domains differed between inpatients and outpatients. These authors [10] observed a positive correlation between daily minutes of moderate physical activity and Processing Speed, although we found no significant association. Moreover, Kimhy et al. [9] reported a positive association between maximum oxygen consumption and neurocognitive function in schizophrenia outpatients. Kimhy et al. [20] and Ho et al. [21] has conducted an RCT of exercise in outpatients with schizophrenia and reported that the aerobic group showed greater improvements in neurocognitive functions than the control (treatment as usual) group. Thus, physical activity appears to differentially relate to the neurocognitive functioning of inpatients and outpatients, with clear benefits in outpatients but not in inpatients.

In contrast to our results, Oertal et al. [22] reported beneficial effects of aerobic exercise on neurocognitive function in inpatients with acute phase schizophrenia. However, the discrepancy with our results may stem from the difference in symptom severity, as the PANSS negative symptom score in the study of Oertal et al. [22] was only 15.55 ± 30.1, markedly lower than in our study (25.7 ± 5.8). This suggests that the relationships between better neurocognitive function and higher physical activity may not emerge in inpatients with schizophrenia, indicating severe negative symptoms. The participants in the current study were non-remitted and generally more severely affected by schizophrenic symptoms. The mean antipsychotic dose in the study by Kimhy et al. [9] was 351.38 ± 291.77 mg, markedly lower than that of our inpatient group (1185.0 ± 890.4 mg). Moreover, the participants in the current study were older (59.4 ± 12.1 years old). Thus, the participants in this study are likely more to be more treatment resistant. We suggest that the features of treatment-resistance may influence the relationships between physical activity and several neurocognitive domains.

These negative correlations with high physical activity level may be explained by the following two points: the particularly strong negative correlations with Complex Attention and Simple Attention domains in inpatients and the basal nucleus.

Attentional functions are the foundations for other neurocognitive domains. Briefly, if attentional function deteriorates, tests for other neurocognitive domains cannot be performed to the participants’ full ability. This may account for the observed reductions in NCI and domain scores for Composite Memory, Verbal Memory, and Visual Memory with longer time spent in vigorous exercise.

In contrast, the basal nucleus is associated not only with moving but also with attention and learning [23]. Abnormality of the basal nucleus in schizophrenia has been reported [24]. Furthermore, anatomical differences in the basal nucleus between treatment-responsive and treatment-resistant patients have been reported in a recent systematic review [25]. Therefore, when patients with treatment-resistant schizophrenia do hard physical activity, it is thought that disability related to treatment resistance decreases the ability to deal with attention and learning, via basal nucleus hyperactivity. However, given the cross-sectional study design, the relationship can also be explained by oppositional interpretation; inpatients with worse attentional deficits tend to bustle. Because there is no evidence that can conclude the mechanism underlying the relationships, we can conclude the causal relationships between high physical activity and worse attentional function.

Therefore, a future study is needed to examine the relationship between physical activity and neurocognitive function, separating inpatients from outpatients, and furthermore to reveal difference in effects of physical activity on brain activity using interventional study design.

This study’s design has several limitations. First, the sample size was small, and convenience sampling was used; therefore, the results will not be generalizable. In comparison with a large-scale study in Japan, the average age of this sample (59.4 ± 12.1 years) was relatively older than that of the large scale study (51.9 ± 11.6 years) [8, 21, 26]. Advanced age may affect the relationship between physical activity and neurocognitive function. Thus, future studies are warranted to investigate a larger sample of patients, using probability sampling, to ensure generalizability.

Second, parametric analysis was performed in this study to examine the relationships between physical activity and neurocognitive function after controlling for other confounding factors (demographics and psychiatric symptoms), although the sample size was limited. Third, the study was cross-sectional; therefore, no causal correlation could be established. Fourth, as these patients become more treatment resistant, future studies are required to determine the influence of physical activity levels on neurocognitive function in treatment-responsive inpatients. Third, the clinical significance of the relationship between physical activity and neurocognitive function could not be determined in this study.

However, the strength of this study was that the differences in the relationships between physical activity and neurocognitive function among inpatients and outpatients were examined, controlling for several confounding factors, which commonly affect data, such as demographics and psychiatric symptoms, in a clinical setting. Further studies are needed controlling for other confounding factors to investigate the differences between in-patients and out-patients.

## Conclusion

The correlation between physical activity and neurocognitive function were different, according to neurocognitive domains and treatment sites. In separated analysis according to the treatment sites (inpatients and outpatients), physical activity has only a negative correlation with neurocognitive function in inpatients, although it had only a positive correlation with neurocognitive function in outpatients. Therefore, when correlations between physical activity and neurocognitive function are examined, the analysis should be performed, separating inpatients particularly treatment-resistance patients from outpatients.

## Additional file

Additional file 1: Table S1. The correlation among Demographics, Psychiatric symptoms, and Neurocognitive functions in inpatients group (n = 16). **Table S2.** The correlation among Demographics, Psychiatric symptoms, and Neurocognitive functions in outpatients group (n = 13). (XLSX 26 kb)
